# Integrated arts engagement as preventive infrastructure for active and healthy aging: a conceptual and policy framework

**DOI:** 10.3389/fpubh.2026.1896804

**Published:** 2026-08-07

**Authors:** Feng Li, Haoyu Suo, Sinuo Chen, Chengmeng Zhang

**Affiliations:** 1 School of Music, Anyang Normal University, Anyang, Henan, China; 2 Social Development Research Institute, National Development and Reform Commission, Beijing, China; 3 Art and Technology/Sound Practices Department, School of the Art Institute of Chicago (SAIC), Chicago, IL, United States; 4 Institute of Population Research, Peking University, Beijing, China

**Keywords:** arts and health, healthy aging, integrated community arts engagement, preventive public health, social prescribing

## Abstract

Population aging is changing the work of public health. Longer lives require not only medical treatment, but also support for functional ability, social connection, meaning, participation, and everyday wellbeing. Arts engagement is increasingly discussed in relation to health, yet arts activities often remain treated as cultural or recreational provision rather than as part of preventive public health systems. This conceptual article develops the idea of integrated community arts engagement as a framework for understanding how arts participation can contribute to healthy aging. The framework draws on literature in arts and health, healthy aging, social prescribing, community participation, and health equity. Integrated community arts engagement proposed here refers to community-based arts activities that are meaningful to older adults, accessible in everyday settings, connected to health and social care pathways, supported by local partnerships, and evaluated as part of healthy aging systems. The framework proposes six hypothesized mechanisms and five policy pathways for system integration. Reframing arts engagement as preventive infrastructure does not medicalize the arts. Rather, it recognizes that health in later life is shaped in ordinary social and cultural settings. These pathways are conceptual propositions rather than empirical findings of this article. Integrated community arts engagement may help aging societies build more humane, locally grounded, and culturally responsive approaches to prevention and healthy aging.

## 1 Introduction

Population aging has become one of the major public health issues of the 21st century. In many societies, people are living longer than previous generations. This is a major social achievement. At the same time, longer life does not always mean a longer period of good health, independence, social connection, or meaningful participation. Health systems are therefore facing a broader task. They must not only treat illness in later life, but also support older adults to remain connected, active, valued, and able to live with dignity (1, 2).

The World Health Organization defines healthy aging as the process of developing and maintaining the functional ability that enables wellbeing in older age. This definition is important because it moves attention beyond disease status alone. It asks how people live in relation to their social, physical, and cultural environments. It also points to a central public health question: what kinds of everyday resources help older adults maintain wellbeing before problems become acute? (1, 3) Many challenges in later life are not produced only in clinical settings and cannot be solved by clinical services alone. Loneliness, reduced mobility, bereavement, ageism, loss of confidence, and shrinking social roles often unfold in ordinary life. These experiences are shaped by housing, transport, family support, income, neighborhoods, social networks, and opportunities for participation. Prevention in later life therefore needs to be understood not only as risk reduction, but also as the creation of social and cultural conditions that help people age well (4–6).

The concept of active aging provides an important bridge between healthy aging and preventive public health. Active aging is commonly understood as a process of optimizing opportunities for health, participation, security, and lifelong engagement as people grow older (7). It does not refer only to physical activity or paid work (8). Rather, it includes a wide range of social, cultural, civic, educational, creative, and relational activities through which older adults remain involved in community life (9).

From a preventive perspective, active aging is important because it shifts attention from responding to decline after it occurs to sustaining capacities, relationships, and meaningful roles before social and functional losses become severe. Participation itself can be understood as a preventive resource (10). It may support routine, confidence, social recognition, and a sense of purpose, all of which are closely related to wellbeing in later life (11). Arts engagement could be understood as one specific but highly integrative domain of active and healthy aging (12). Arts engagement, including music, dance, storytelling, visual arts, community singing, calligraphy, and other locally meaningful cultural practices, could offer older adults' ways to move, remember, learn, express, meet others, and remain visible in social life. It connects older adults' participation in cultural life with social interaction, emotional expression, creativity, embodied activity, memory, and community belonging (13). These activities are usually not entered through the language of illness. People often take part because they are enjoyable, familiar, culturally meaningful, or socially valued. This makes arts engagement a distinctive route into health promotion. It can support wellbeing without first asking older adults to define themselves as patients (14–16). In this sense, arts engagement is not only a cultural or recreational activity, but also a potential preventive resource through which older adults may maintain connection, meaning, and visibility in everyday life.

This perspective article argues that arts engagement should be understood as part of the preventive infrastructure of healthy aging. The aim is not to turn every arts activity into a health intervention. Nor is it to suggest that arts engagement can replace medical care, social care, or income support. Rather, the article asks how public health systems can better recognize, connect, support, and evaluate the contribution of arts engagement to later life wellbeing (17, 18). To develop this argument, the article proposes the concept and framework of Integrated Community Arts Engagement. This concept offers a way to move beyond general claims that the arts are good for health. It focuses instead on the conditions under which arts participation becomes accessible, meaningful, sustained, and connected to wider healthy aging systems (19–21). As a conceptual and policy framework piece, this perspective article does not report original empirical data or test the proposed framework. The framework was developed through a narrative synthesis of literature on arts and health, healthy aging, social prescribing, community participation, creative health, and health equity, combined with policy-oriented conceptual analysis. The proposed mechanisms and outcomes should therefore be understood as hypothesized pathways that require future empirical testing.

## 2 From arts participation to preventive infrastructure

The arts and health field has grown rapidly in recent years, with increasing attention to arts engagement involving older adults. A broad body of work has linked arts engagement with mental wellbeing, quality of life, social connection, cognitive function, and reduced loneliness (22). These findings are especially relevant for older adults, because later life often involves changes in social networks, daily routines, bodily capacity, and identity (17, 23).

Arts engagement can take many forms. Some are receptive, such as attending concerts, visiting museums, watching theater, or listening to music. Others are participatory, such as singing, dancing, painting, making crafts, acting, writing, or telling stories (24). The boundary between these forms is not fixed. A museum visit may involve memory, discussion, movement, and social contact. A music session may involve listening, singing, rhythm, emotion, and shared attention. A craft group may involve manual skill, concentration, conversation, and cultural memory (15).

For healthy aging, the value of arts engagement lies partly in this combination of dimensions. Many health promotion activities target one domain at a time, such as exercise, cognition, or mental health (25). Arts activities often bring several domains together. A dance group may support movement, memory, confidence, and friendship. A choir may support breath, voice, discipline, emotion, and belonging. A visual arts workshop may support attention, creativity, identity, and conversation (26, 27).

This combined quality is important because aging is not experienced in separate categories. Physical health, mental wellbeing, social connection, cultural identity, and sense of purpose are often closely linked. Arts engagement may therefore work not as a single treatment, but as a set of social and cultural practices that support several aspects of wellbeing at once.

At the same time, the evidence should still be interpreted with care. Much research in arts and health is observational, and participation in cultural activities is shaped by education, income, health status, mobility, and local opportunity (28). Selection bias is therefore a major concern, as older adults who are healthier, wealthier, more mobile, better educated, or already socially connected may be more likely to participate in arts activities. It is also difficult to establish causality, because arts engagement may both influence and be influenced by wellbeing, confidence, and social participation. Causal claims should therefore be cautious. Arts engagement is also a broad and varied category. Individual museum visits, group singing, dance, storytelling, crafts, digital arts, and community theater may differ in intensity, duration, facilitation, social interaction, cultural meaning, and participant population. Their possible contributions to healthy aging are therefore unlikely to be identical (27, 29). Some activities may be more strongly linked to social connection, while others may be more relevant to physical activation, memory, identity, or cultural belonging. These limitations also have policy implications. Poorly designed or inaccessible programmes may mainly reach older adults who already have transport, time, confidence, cultural capital, and local opportunities (30). In such cases, arts engagement may reproduce exclusion rather than reduce it. Evidence generated in one cultural or health-system context also cannot be assumed to transfer directly to another. However, this does not weaken the policy relevance of the field. Public health often works with complex social practices where simple causal pathways are hard to isolate. The more useful question is not whether arts engagement can contribute to one uniform effect. It is how, for whom, in what settings, and under what system conditions arts engagement can support healthy aging (31).

This article therefore treats arts engagement as a social and cultural resource with potential public health value. Its contribution depends on access, quality, continuity, cultural meaning, and the way it is connected to local systems. This is why a framework for integration is needed (32, 33).

## 3 Conceptual frameworks: integrated community arts engagement

This article proposes integrated community arts engagement as a conceptual and policy framework for healthy aging. The term refers to arts activities that are located in community life, meaningful to older adults, accessible in everyday settings, connected to health and social care pathways, and supported by local policy and organizational arrangements (34). The word “integrated” is central. Many arts activities for older adults already exist, but they often remain separate from health and social care systems. They may be offered by cultural institutions, voluntary organizations, libraries, community centers, religious groups, care homes, neighborhood groups, or informal associations. These activities may support wellbeing, but their contribution is often not visible to public health planning. Older adults who could benefit may not know they exist. Health professionals may not have clear routes for referral. Programmes may depend on short term funding or a small number of committed organizers. Evaluation may count attendance but miss changes in confidence, routine, belonging, or social participation (24, 25). Integrated community arts engagement addresses this gap. It does not mean making all arts activities clinical. It means building clearer connections between arts participation and the wider conditions that support healthy aging. These connections include policy recognition, community referral, stable provision, suitable evaluation, and shared governance across sectors. The concept therefore sits between culture, public health, social care, and everyday community life.

The originality of the proposed framework here lies in its system-level orientation. Social prescribing models have usefully highlighted referral from clinical or community settings to non-medical resources, but referral alone does not explain how arts activities become sustained, accessible, culturally meaningful, and accountable parts of healthy aging systems. Creative health frameworks have advanced the broader health value of arts and creativity, but they often remain broad in scope. WHO healthy aging frameworks define functional ability and supportive environments, but they do not specify how community arts practices can be organized as part of such environments. Arts-in-health intervention models often focus on specific programmes or populations. By contrast, integrated community arts engagement is proposed here as a preventive infrastructure framework that links community arts resources, hypothesized mechanisms, system integration functions, equity conditions, and healthy aging outcomes.

### 3.1 The framework has three connected levels

The first level concerns arts engagement resources, including receptive, participatory, formal, informal, institution-based, and community-led practices (35). These resources may vary across cultural and local contexts. A broad definition is needed because older adults do not engage with the arts in only one way. Their participation is shaped by ability, income, habit, language, culture, place, and previous life experience (14, 16, 27).

The second level concerns health supporting mechanisms. Six mechanisms are especially relevant: social connection, emotional expression, physical activation, cognitive stimulation, identity and meaning, and cultural belonging. These mechanisms are not separate channels. They often work together. A choir may support breathing, memory, social ties, emotion, and belonging at the same time. A dance group may combine movement, rhythm, confidence, social identity, and routine. A museum programme may involve memory, aesthetic experience, conversation, and intergenerational exchange (15, 17, 25).

The third level concerns system integration. For arts engagement to become part of preventive public health, it needs pathways that make participation visible, reachable, sustained, and fair. These pathways include policy recognition, community referral, sustainable provision, suitable evaluation, and cross-sector governance. Without these pathways, arts activities may remain valuable but fragile. They may benefit those who already have cultural confidence and social access, while leaving out older adults who face poverty, disability, isolation, language barriers, or rural distance (18, 36, 37).

The framework is also shaped by moderating conditions. These include income, education, disability, mobility, sensory impairment, gender, ethnicity, migration history, rurality, digital access, caring responsibilities, previous arts experience, and local cultural infrastructure. These conditions are not background details. They shape who can take part, how participation is experienced, and whether arts based healthy aging work reduces or reproduces inequality (4, 38, 39).

**Figure 1:**
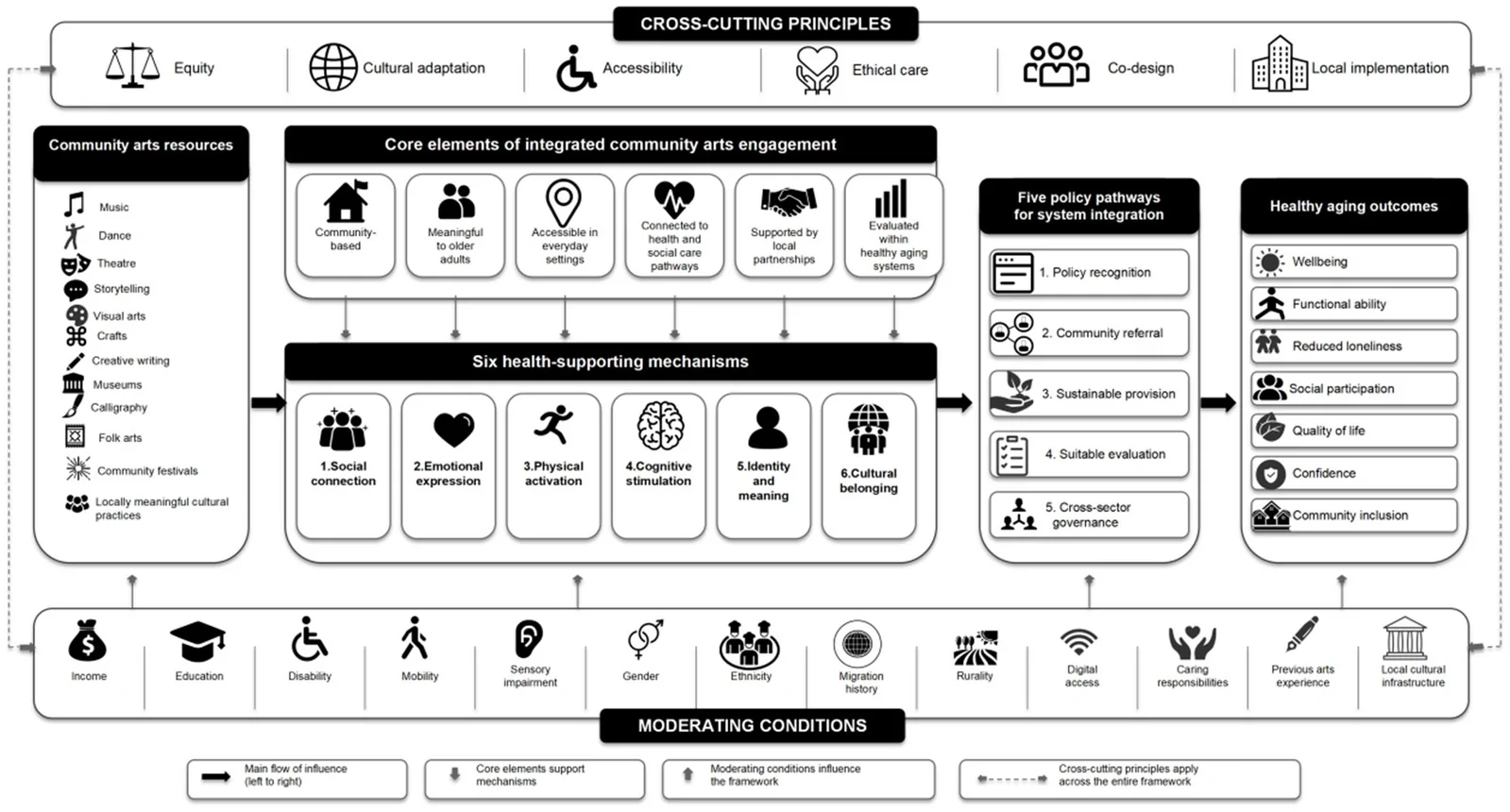
Integrated community arts engagement as preventive infrastructure for healthy aging.

Figure 1 represents the framework as a conceptual map rather than as a tested causal model. The figure should be read from left to right, beginning with community arts resources and moving through the core elements of integrated community arts engagement, the six health-supporting mechanisms, the five policy pathways for system integration, and the proposed healthy aging outcomes. The cross-cutting principles and moderating conditions indicate that the pathway is not automatic. Access, culture, disability, mobility, income, digital inclusion, previous arts experience, and local cultural infrastructure may shape whether arts engagement becomes meaningful, sustained, and equitable. On the left are community arts resources. In the middle are the six mechanisms. These mechanisms are connected to system pathways that determine whether arts engagement remains isolated or becomes part of preventive infrastructure. On the right are healthy aging outcomes, including wellbeing, functional ability, reduced loneliness, social participation, quality of life, confidence, and community inclusion. Across the framework are cross cutting principles: equity, cultural adaptation, accessibility, ethical care, and local implementation (15, 25, 26).

### 3.2 Mechanisms linking arts engagement to healthy aging

The six mechanisms proposed in this framework are derived from a synthesis of arts-and-health literature, healthy aging theory, social prescribing research, and the authors' conceptual interpretation of community-based cultural participation. They should be understood as hypothesized and overlapping pathways rather than as separate or empirically proven effects of this article. In practice, one arts activity may activate several mechanisms at the same time. For example, group singing may support social connection, emotional expression, breath and voice, memory, and cultural belonging. Dance may be more strongly linked to physical activation, rhythm, bodily confidence, and group participation. Storytelling and theater may be especially relevant to identity, memory, agency, and shared meaning. Calligraphy, crafts, and visual arts may involve attention, fine motor activity, creativity, and cultural continuity. These examples show that the framework is not intended to assign each art form to a single mechanism, but to help researchers and practitioners consider which mechanisms are most likely to be activated in each context.

The first mechanism is social connection. Many older adults experience shrinking social networks due to retirement, bereavement, illness, relocation, or reduced mobility (40). Group based arts activities can create repeated opportunities for contact. They may also reduce the pressure of direct social interaction, because people meet around a shared activity. Singing, painting, dancing, or attending a cultural event gives participants something to do together, something to talk about (41). This can be valuable for older adults who may feel uncomfortable joining activities framed as loneliness support (5).

The second mechanism is emotional expression. Later life can bring grief, anxiety, frustration, pride, joy, and changes in self-image. Arts activities provide ways to express emotion without relying only on direct verbal explanation. Music, images, movement, theater, and storytelling can make room for feelings that may be difficult to name. This does not mean that all arts activities are therapy in a clinical sense. It means that they can offer everyday forms of emotional processing and support (15, 24). Music, storytelling, theater, and visual arts may be especially relevant here because they allow feeling, memory, and personal experience to be expressed indirectly or symbolically.

The third mechanism is physical activation. Some arts activities may support bodily confidence and gentle activity involve movement, breath, posture, coordination, rhythm, voice, or manual skill. This matters because physical inactivity in later life is not only a matter of motivation. It is also shaped by fear of falling, pain, lack of suitable spaces, embarrassment, and the feeling that exercise programmes are not socially or culturally appealing. Arts based movement can make activity feel less like treatment and more like participation (17, 41, 42). Physical activaties may activate the body in different ways, ranging from balance and coordination to breath, voice, posture, and fine motor movement.

The fourth mechanism is cognitive stimulation. Arts engagement can involve attention, learning, memory, imagination, sequencing, and interpretation. This form of stimulation should not be understood only as individual cognitive training, because cognitive aging is also shaped by neighborhood ecologies, cultural infrastructures, and opportunities for social participation, and cognitive support is closely connected with relational reconnection and social support (43, 44). A theater group may require role taking and recall. A music session may involve rhythm, listening, and memory. A museum programme may invite observation and discussion. A craft group may involve planning and manual skill. These activities may involve attention, memory recall, learning, sequencing, and interpretation, support cognitive engagement in ways that are enjoyable and socially embedded (15, 25, 26).

The fifth mechanism is identity and meaning. Older adults are often described through the language of need, risk, dependence, and care. Arts engagement can make other identities visible: singer, dancer, painter, storyteller, audience member, maker, volunteer, teacher, or cultural bearer. These identities matter because they allow older adults to be recognized as people with histories, tastes, skills, and contributions (32, 45). The participatory visual arts may be especially useful for supporting narrative identity and a sense of continuing social value.

The sixth mechanism is cultural belonging. Arts practices carry cultural memory and local meaning. Music, dance, craft, opera, poetry, religious song, storytelling, calligraphy, festival practices, and popular cultural forms can connect older adults to language, place, family history, migration experience, and community identity. For minority groups, migrants, rural communities, and people whose cultural forms are often underrepresented in formal institutions, this mechanism may be especially important (3, 16, 33). This mechanism is especially important when arts activities are grounded in local language, regional traditions or other familiar cultural forms.

Taken together, these six mechanisms explain why arts engagement is relevant to active aging and healthy aging without reducing it to a single therapeutic function. Its value lies in the way arts practices may connect body, mind, emotion, social life, identity, and culture (33, 42, 45). The mechanisms should therefore be used as an analytical guide for implementation and evaluation, rather than as a fixed classification of art forms. In practice, the same activity may support several mechanisms, and the same mechanism may be activated through different cultural practices depending on local context, participant preference, and programme design.

## 4 From availability to integration

### 4.1 Integration possibility of arts community engagement

In many communities, arts opportunities for older adults already exist. Libraries, museums, cultural centers, community halls, faith organizations, voluntary groups, care homes, neighborhood associations, and local artists may all provide activities. The problem is not always absence. More often, the problem is weak integration (34, 36, 37).

Health and social care professionals may not know what cultural resources are available. Arts organizations may not have stable links with health services. Older adults may not receive information in accessible forms. Activities may be located far from those with mobility problems. Programmes may be short term or project based. Evaluation may focus on satisfaction while missing deeper questions of confidence, belonging, participation, and cultural meaning (22, 32).

This is why arts engagement should be considered at a system level. A single project may be valuable, but it does not by itself create public health infrastructure. Infrastructure requires visibility, pathways, continuity, quality, and accountability. It also requires attention to who is included and who is left out.

Social prescribing has created one route for connecting people to community activities, including arts-based activities. It can help bridge clinical and community settings. Yet referral alone is not enough. Referral without accessible provision can create disappointment. Provision without cultural fit can reproduce exclusion. Evaluation without attention to meaning can undervalue what participants gain. For arts engagement to contribute to healthy aging, the connections between policy, referral, provision, evaluation, and governance need to be stronger (18, 20).

### 4.2 Five policy pathways for integrated community arts engagement

The first pathway is policy recognition. Healthy aging policies often refer to wellbeing, participation, loneliness, prevention, and community support. Yet cultural participation is not always named as a practical route for achieving these goals. Recognition matters because policy language shapes funding, service design, and professional attention. Arts engagement should be included in healthy aging strategies, loneliness prevention plans, dementia friendly community work, social prescribing frameworks, and local public health planning (21, 34, 46).

The second pathway is community referral. Referral systems should not depend only on individual clinicians who happen to know local programmes. General practitioners, nurses, social workers, link workers, housing providers, cultural organizations, voluntary groups, and older people's services can all help connect older adults to suitable opportunities. Referral should be based on preference and fit. An older adult who enjoys opera may not want the same activity as someone who prefers dance, craft, religious music, gardening, painting, or local storytelling. Referral should open choices rather than impose participation (19, 20, 47, 48).

The third pathway is sustainable provision. Many arts and health projects are funded as pilots. Pilots can be useful, but they do not provide continuity. Older adults may build trust and relationships, only to see a programme end when funding stops. Sustainable provision requires long term commissioning, accessible venues, transport support, low cost or free access, trained facilitators, and links to existing community infrastructure. Digital delivery may help some older adults, especially those with mobility limits or living in remote areas. However, digital options should not replace face to face activity where social contact is the main need (18, 37).

The fourth pathway is suitable evaluation. Standard health evaluation may not fully capture the value of arts engagement. Measures of loneliness, mood, quality of life, physical activity, cognition, and service use can be useful, but they should be combined with qualitative accounts of confidence, belonging, identity, and meaning. Evaluation should also examine process: who attends, who does not, what supports participation, what barriers remain, and how different groups experience the activity. Economic evaluation may be important for policy, but it should not reduce the arts to cost saving. Realist evaluation may be useful because it asks what works, for whom, in what circumstances, and why. This fits the nature of arts engagement, where context matters. A singing group in one community may work because of a trusted local facilitator. A museum programme may succeed because transport and welcome are carefully organized. A digital arts activity may help homebound older adults but exclude those with limited digital access. Evaluation should make these conditions visible (37–39).

The fifth pathway is cross-sector governance. Arts engagement sits between health, culture, social care, local government, voluntary organizations, and community life. If responsibility is unclear, programmes can fall between sectors. Cross-sector governance may include shared commissioning, local directories, referral agreements, joint training, and common evaluation principles. Older adults should also be involved in design and evaluation. Without their involvement, programmes risk reflecting institutional assumptions rather than lived experience (34, 36). Co-design is central to this governance pathway and could be understood as a process of value co-creation. In integrated community arts engagement, value is not produced by arts programmes alone, nor by health systems alone. It is co-created through the participation of older adults, arts practitioners, community organizations, health and social care providers, and local authorities (49, 50). Older adults should therefore not be positioned only as recipients of arts-based support. They should be involved in identifying needs, selecting culturally meaningful art forms, shaping referral routes, defining access barriers, and deciding what outcomes should be evaluated (51). Co-design could help ensure that arts engagement is not imposed as a top-down health intervention, but developed as a shared practice through which older adults contribute to community life, cultural continuity, and local wellbeing.

To make these policy pathways more operational, Table 1 summarizes their core functions, possible implementation examples, and risks to avoid.

**Table 1:** Policy pathways for integrated community arts engagement.

Policy pathway	Core function	Implementation examples	Risks to avoid
Policy recognition	Makes arts engagement visible within healthy aging and prevention policy	Include arts engagement in healthy aging plans, loneliness prevention strategies, dementia-friendly community work, social prescribing frameworks, and age-friendly community planning	Symbolic recognition without funding, delivery structures, or accountability
Community referral	Connects older adults with suitable and meaningful arts opportunities	Use link workers, community health centers, social workers, older people's organizations, cultural directories, and preference-based matching	Referral without accessible provision; treating referral as prescription rather than choice
Sustainable provision	Supports continuity, access, and programme quality beyond short-term projects	Long-term commissioning, accessible venues, transport support, free or low-cost access, facilitator training, and links with local cultural infrastructure	Short-term pilot dependency; unstable staffing; exclusion of older adults with mobility or income barriers
Suitable evaluation	Captures both health-related and cultural value	Mixed-methods evaluation, realist evaluation, wellbeing and participation measures, qualitative accounts of meaning, equity monitoring, and participant-defined outcomes	Reducing arts engagement to clinical metrics or cost savings alone
Cross-sector governance	Creates shared responsibility and supports co-design across health, culture, social care, local government, community organizations, and older adults	Shared directories, referral agreements, joint training, partnership boards, co-design workshops with older adults, participatory evaluation, and value co-creation processes	Fragmented responsibility; unclear accountability; programmes designed without older adults' voices

### 4.3 Illustrative contexts: community arts, aging, and local governance in China

China provides a useful illustrative context for this framework because it brings together rapid population aging, growing pressure on family care, and community-based support, strong neighborhood-level governance, and a rich everyday ecology of older adults' cultural participation. The relevance of China does not lie only in the size of its older population. It also lies in the fact that many forms of arts engagement already take place in ordinary community settings, including public squares, community centers, senior universities, neighborhood committees, cultural institutions, and informal groups (1, 2). This makes China a useful case for considering how arts engagement can move from informal cultural participation toward a more visible part of healthy aging systems.

In many Chinese communities, older adults already take part in activities such as community singing, square dancing, calligraphy, painting, opera, folk music, handicrafts, cultural festivals, and programmes organized through community centers and senior universities. These practices are not only leisure activities. They may also support routine, movement, peer contact, cultural continuity, public visibility, and a sense of belonging. Square dancing, for example, can be understood not only as physical activity, but also as a form of social organization, neighborhood presence, rhythm, identity, and mutual support (52). Calligraphy and painting groups may support concentration, cultural continuity, and intergenerational exchange. Community singing and opera groups may connect memory, language, emotion, and local identity.

At the same time, China should not be treated as a homogeneous context. Urban and rural communities differ in cultural infrastructure, public space, transport, service capacity, and access to organized arts activities. Regional cultures also shape which art forms are familiar and meaningful to older adults. Socioeconomic inequality may affect who can access senior universities, cultural centers, digital arts activities, or fee-based community programmes. Digital access is also uneven, and older adults with limited digital skills may be excluded from online information, registration systems, or hybrid cultural activities. These differences show why integrated community arts engagement needs to be understood through local conditions rather than treated as a standard model that can be applied uniformly across all settings (37, 39).

However, these activities are not always understood as part of public health. They may be treated as leisure, culture, or informal self-organization. This can limit their integration with healthy aging policy, primary care, community health services, and social support systems. A framework approach would not require turning these practices into clinical interventions. Instead, it would ask how existing cultural participation can be supported more fairly, connected more effectively, and evaluated more appropriately (18, 19).

For example, community health centers could work with cultural centers and older people's organizations to identify local arts opportunities. Community workers could help socially isolated older adults find suitable groups. Local governments could support accessible venues and reduce conflicts over public space. Local governments could support accessible venues, transport arrangements, and age-friendly public spaces, while also reducing conflicts over shared public space. Senior universities and cultural institutions could act as local hubs for arts participation, intergenerational learning, and cultural continuity. Such an approach would recognize that healthy aging is produced through ordinary social and cultural life, not only through health services.

The Chinese context also highlights the importance of cultural adaptation. A model based only on Western social prescribing may not fit all settings. Local governance structures, family expectations, community norms, and cultural forms need to be considered. This is also true beyond China. Any attempt to integrate arts engagement into healthy aging systems should begin with local cultural practices and older adults' own preferences, rather than importing a fixed programme model from another health or cultural system.

## 5 Equity, ethics, and cultural adaptation

Equity should be central to integrated community arts engagement. Cultural participation is often unevenly distributed. Older adults with higher income, higher education, better mobility, and stronger social networks may be more likely to attend arts activities. Those facing poverty, disability, rural isolation, language barriers, caring responsibilities, or minority status may face greater barriers. If policy simply expands arts engagement without addressing access, it may benefit those already advantaged (4, 38).

Practical barriers include cost, transport, venue access, information, digital literacy, sensory impairment, and fear of not belonging. Cultural barriers are equally important. Some older adults may feel that certain arts spaces are not for people like them. Others may not recognize their own cultural practices in official programmes. A broad definition of arts engagement is therefore needed. It should include everyday, community based, popular, traditional, minority, and informal cultural forms, not only formal arts institutions (3, 6, 39).

The proposed framework could guide future research and evaluation. Because arts engagement is complex, no single method is sufficient. Mixed methods are especially suitable. Quantitative measures can assess loneliness, wellbeing, mood, quality of life, physical activity, cognitive engagement, or service use. Qualitative interviews and observations can explore meaning, identity, confidence, relationships, and cultural fit. Participatory evaluation can involve older adults in defining what counts as success (15, 17).

Short term evaluation might examine attendance, enjoyment, mood, perceived connection, and willingness to return. Medium term evaluation could examine sustained participation, new relationships, confidence, routine, and perceived social support. Longer term evaluation might consider functional ability, quality of life, reduced isolation, community inclusion, and possible changes in use of health or social care services. These outcomes should be interpreted carefully. Arts engagement is one part of a wider system, not a single cause of healthy aging (21, 23, 53).

Implementation research is also needed. Studies could compare different referral models, such as clinician referral, link worker referral, community outreach, and self-referral. Research could examine what makes partnerships between health and cultural sectors work well. It could also study why some programmes fail to reach those most at risk of isolation. Equity focused evaluation should track who participates, who drops out, and who never enters the pathway (39, 48, 54).

Ethical issues also require attention. Arts activities can involve emotion, memory, personal stories, and vulnerability. Facilitators need to respect consent, privacy, boundaries, and the right not to participate. Older adults should not be pressured to join activities because professionals believe participation is good for them. Care is also needed when working with people living with dementia, grief, trauma, or social isolation (55). The aim should be supportive participation, not the extraction of personal stories or the performance of wellbeing for funders (36, 38). From an ethical perspective, co-design and value co-creation also help prevent the instrumentalization of older adults' participation. They recognize older adults as contributors to cultural and community value, rather than as passive subjects whose participation is measured only through health outcomes.

There is also a broader ethical concern about instrumentalizing the arts. If the arts are valued only because they reduce loneliness or save health costs, their cultural and human value may be narrowed. A balanced framework should recognize both intrinsic and instrumental value. The arts may activate health, but they also matter because they help people express, remember, imagine, and belong. This balance is especially important in later life, when older adults are often reduced to categories of burden, risk, or need (16, 34).

## 6 Conclusion

Healthy aging requires more than longer survival. It requires environments that could support participation, connection, function, meaning, and dignity. Arts engagement offers one route toward these goals because it works through everyday forms of social and cultural life. It can help older adults move, remember, express, learn, meet others, and remain visible as people with histories and contributions (14, 17).

This article has proposed integrated community arts engagement as a conceptual and policy framework for understanding this contribution. The framework links community arts resources, health supporting mechanisms, system pathways, and healthy aging outcomes. It also places equity, cultural adaptation, ethical care, and local implementation at the center. Reframing arts engagement as preventive infrastructure does not reduce the arts to medicine. Instead, the framework encourages a move beyond broad claims that the arts are good for health. The arts are not a decorative addition to healthy aging policy. They are one way of making longer lives more connected, meaningful, and livable. This framework aims to emphasize that health in later life is shaped in ordinary places and practices, aging societies need public health systems that can work with these resources (18, 19, 21).

This article has several limitations. First, it is a conceptual and policy framework article and does not test the proposed framework empirically. The mechanisms and outcomes should therefore be read as hypothesized pathways rather than demonstrated effects. Second, much of the evidence on arts engagement and health remains observational, making causal interpretation difficult. Third, the framework may not apply equally across cultural, socioeconomic, rural, urban, and health-system contexts. Fourth, arts engagement may not benefit all older adults equally. Without attention to cost, transport, disability access, language, digital inclusion, and cultural relevance, programmes may reproduce existing inequalities. Fifth, the proposed mechanisms and policy pathways require future empirical testing through mixed-methods, implementation research, realist evaluation, and equity-focused studies.

For practice, the framework suggests that arts-based healthy aging work should be locally grounded and co-designed. Practitioners should begin by mapping existing cultural resources and listening to older adults' preferences, but they should also involve older adults in shaping programme aims, selecting meaningful activities, identifying access barriers, and defining what counts as success (37). In this sense, older adults are not only participants in arts engagement. They are co-creators of social, cultural, and health-related value within their communities.

For policy, the framework points to the need for shared responsibility. Arts engagement cannot be sustained if it is treated as an optional extra or left to short term grants. Public health, culture, social care, local government, and community organizations all have a role. Policy should support long term partnerships, referral infrastructure, accessible spaces, and evaluation that respects both health and cultural value. This does not mean that all arts activities should become public health programmes. It means that public health systems should recognize cultural participation as one of the conditions that can help people age well (46, 53).

Although this article focuses on arts engagement, the broader logic may also be relevant to other domains of active aging. Physical activity, volunteering, lifelong learning, civic participation, intergenerational exchange, digital participation, and community mutual aid may also become preventive resources when they are meaningful, accessible, sustained, and connected to local systems of support. In this sense, the framework developed here is domain-specific but not conceptually closed. It shows how one active aging domain may be analyzed through resources, mechanisms, system pathways, equity conditions, and outcomes. Future work could examine whether similar preventive infrastructure frameworks could be developed for other domains of active aging.

Future studies should specify mechanisms, contexts, and populations. They could further examine how different art forms operate, how social and cultural meanings shape outcomes, and how inequalities influence access. Research should also avoid treating older adults as a single group. Age, gender, class, ethnicity, disability, rurality, and previous cultural experience all matter (22, 24, 54).

## Data Availability

The original contributions presented in the study are included in the article/supplementary material, further inquiries can be directed to the corresponding author.
